# Formation and Thermal Stability of the ω-Phase in Ti–Nb and Ti–Mo Alloys Subjected to HPT

**DOI:** 10.3390/ma15124136

**Published:** 2022-06-10

**Authors:** Anna Korneva, Boris Straumal, Alena Gornakova, Askar Kilmametov, Łukasz Gondek, Lidia Lityńska-Dobrzyńska, Robert Chulist, Małgorzata Pomorska, Paweł Zięba

**Affiliations:** 1 Institute of Metallurgy and Materials Science, Polish Academy of Sciences, 30-059 Krakow, Poland; l.litynska@imim.pl (L.L.-D.); r.chulist@imim.pl (R.C.); m.pomorska@imim.pl (M.P.); p.zieba@imim.pl (P.Z.); 2 Osipyan Institute of Solid State Physics of the Russian Academy of Sciences, 142432 Chernogolovka, Russia; straumal@issp.ac.ru (B.S.); alenahas@issp.ac.ru (A.G.); arkilmametov@gmail.com (A.K.); 3 Faculty of Physics and Applied Computer Science, AGH University of Science and Technology, 30-059 Krakow, Poland; lgondek@agh.edu.pl

**Keywords:** Ti alloys, high-pressure torsion, in situ high-temperature X-ray diffraction, phase transformations, thermal stability

## Abstract

This paper discusses the features of ω-phase formation and its thermal stability depending on the phase composition, alloying element and the grain size of the initial microstructure of Ti–Nb and Ti–Mo alloys subjected to high-pressure torsion (HPT) deformation. In the case of two-phase Ti–3wt.% Nb and Ti–20wt.% Nb alloys with different volume fractions of α- and β-phases, a complete β→ω phase transformation and partial α→ω transformation were found. The dependence of the α→ω transformation on the concentration of the alloying element was determined: the greater content of Nb in the α-phase, the lower the amount of ω-phase that was formed from it. In the case of single-phase Ti–Mo alloys, it was found that the amount of ω-phase formed from the coarse-grained β-phase of the Ti–18wt.% Mo alloy was less than the amount of the ω-phase formed from the fine α′-martensite of the Ti–2wt.% Mo alloy. This was despite the fact that the ω-phase is easier to form from the β-phase than from the α- or α′-phase. It is possible that the grain size of the microstructure also affected the phase transformation, namely, the fine martensitic plates more easily gain deformation and overcome the critical shear stresses necessary for the phase transformation. It was also found that the thermal stability of the ω-phase in the Ti–Nb and Ti–Mo alloys increased with the increasing concentration of Nb or Mo.

## 1. Introduction

Titanium alloys have a wide application field in various branches of industry (e.g., aviation, mechanical engineering and biomedicine) due to the fact of their low density, low Young’s modulus, high plasticity, good biocompatibility and excellent corrosion resistance [1,2,3]. Ti–Nb and Ti–Mo alloys are promising for application as metallic biomaterials (in particular, as orthopedic implants), because Nb and Mo are nontoxic, nonallergenic and possess a low Young’s modulus similar to natural bone [4,5,6,7,8]. However, these alloys are seldom used due to the fact of their lower strength in comparison with widely applied biocompatible Ti alloys, such as Ti–6Al–7Nb, Ti–5Al–2.5Fe, or Ti–6Al–4V, which possess restricted biocompatibility and relatively high Young’s modulus [1,9]. Using the methods of severe plastic deformation (SPD), it is possible to improve the strength of materials due to the refinement of the microstructure [10,11] and to reduce the Young’s modulus due to the phase transformations caused by SPD [4]. For example, the high-temperature deformation of Grade-2 purity titanium by the equal-channel angular pressing (ECAP) increases its yield strength from 330 to 652 MPa due to the decrease in grain size to a few dozens of nanometers together with an increase in dislocation density [11]. The high-pressure torsion (HPT) of commercially pure titanium (Grade 4) increases the ultimate tensile strength up to 1600 MPa due to the decrease in grain size [12]. For comparison, the strength of titanium alloys with elements nontoxic to the human body, namely, Ti_15_Mo_7_Zr_5_Ta and Ti_20_Mo_7_Zr_5_Ta, obtained by an arc melting method, are approximately 710 and 1410 MPa, respectively. However, the elongation of these alloys only reached 4.7% [13]. 

Many properties of Ti-based alloys are connected with allotropic transformations. Titanium alloys possess two stable phases, namely, a low-temperature α-phase, with a hexagonal close-packed (hcp) structure, and a high-temperature β-phase, with a body-centered cubic (bcc) structure, as well as three metastable phases (i.e., an α′-martensite with a hexagonal closely packed lattice, an α″-martensite with orthorhombic structure and a ω-phase with a hexagonal structure) [14]. The α′- and α″-martensite phases form by quenching of the β-phase and depend on the chemical composition of the alloys. The ω-phase can be obtained as a result of slow quenching of the β-phase (ω_athermal_), during isothermal aging of the β-phase (ω_isothermal_) or under high-pressure treatment of the β/α-phases, and can be retained after pressure release [4,15,16]. In Ti-based alloys, the high-pressure ω-phase forms more easily from the β-phase during SPD and also from the α-phase at hydrostatic pressures between 2 and 12 GPa [17,18,19], depending on the experimental technique, pressure environment and alloying additions. For example, it was found that even a 0.1 rotation of HPT deformation under 7 GPa and a rotation rate of 1 rpm of the Ti-4wt.%Fe alloy in the β-state led to the formation of 90% of the ω-phase [20]. On the other hand, an increase in the pressure from 3 to 6 GPa over five revolutions and a rotation rate of 1 rpm of commercial purity α-Ti caused an increase in the volume fraction of the ω-phase from 10 to 70%. In addition, an increase in the number of HPT revolutions from ½ to 1 and then to 5 and 10 at a pressure of 6 GPa and a rotation rate of 1 rpm also increased the volume fraction of the ω-phase from 55 to 65, 70 and 90%, respectively [17].

The SPD-induced α→ω and β→ω are martensitic transformations (diffusionless), being promoted by shear strain and alloying of Ti with β-stabilizers (e.g., Fe, Co, Nb, Ni, Mo and V). The solubility of β-stabilizers is minimal in the α-phase and quite high (up to 20 wt.%) in the β-phase. The following question arises: How does the addition of various β-phase stabilizers influence the nature of the α→ω and β→ω phase transformations? The formation of the ω-phase in Ti is related to their specific electronic structure, which is characterized by the relationship that exists between the occupied narrow *d* bands and the broad *sp* bands. Under the applied pressure, the *sp* bands rise faster in energy, causing electrons to be transferred to the *d* bands [21]. This process is known as the *s-d* transition, and it governs the structural properties of transition metals. As β-stabilizers are mostly *d*-electron rich transition elements, this type of alloying gives rise to *d*-electron concentration and can, therefore, provide an additional driving force for the α→ω transformation. In other words, alloying with β-phase stabilizers can be considered as the pressure equivalent for the α→ω phase transformation. Hennig [22] concluded from ab initio calculations employing the density functional theory that alloying with β-stabilizer elements should lead to a decrease in the onset pressure of the α→ω transformation. However, there are no systematic experimental investigations confirming this prediction. Furthermore, the alloying of Ti with β-stabilizers with different solubilities in Ti offers new possibilities for the experimental studies of the α→ω phase transformation.

There are some works dedicated to HPT-induced α→ω [17,23,24,25], β→ω [20,26] and α′→ω [27] transformations and the thermal stability of the SPD-induced ω-phase [17,27,28,29] in pure Ti [17,23,24] as well as in Ti–Fe [20,28,30] and Ti–Co [25,29] alloys. However, there is a lack of systematic studies on the influence of microstructural morphology (i.e., the shape and size of grains) and phase composition on the formation of the ω-phase, especially in Ti–Nb and Ti–Mo alloys subjected to HPT deformation. The novelty of this paper is that it answers the question: How does the amount of the alloying component, different combinations of phase composition (α, β or α′) and the grain size of the initial microstructure affect ω-phase formation, and what is the ω-phase’s thermal stability dependent on? The transformation to the ω-phase at high pressures and shear deformation raises a number of scientific and engineering issues mainly because the ω-phase is fairly brittle compared to the α-phase, which may significantly limit the use of Ti in high-pressure applications [31]. Hence, knowledge on ω-phase thermal stability is needed to determine the conditions for the thermo-mechanical treatment of Ti alloys in order to modify their mechanical properties. Thus, the microstructure, phase transformations (such as α→ω, β→ω and α′→ω) and thermal stability of the HPT-induced ω-phase in deformed Ti–2wt.% Nb, Ti–20wt.% Nb, Ti–2wt.% Mo and Ti–18wt.% Mo alloys were studied in the current work.

## 2. Material and Methods

**Figure 1:**
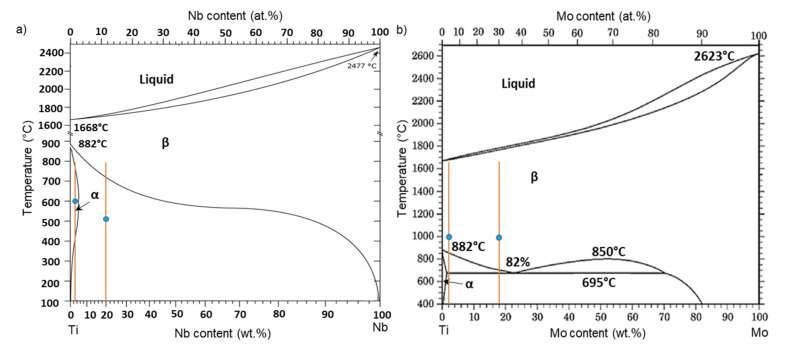
Ti–Nb (**a**) and Mo–Ti (**b**) phase diagrams constructed based on the data [14,32]. Vertical lines and blue circles indicate the phase compositions and the annealing temperatures of the examined alloys, respectively.

The Ti–3wt.% Nb, Ti–20wt.% Nb, Ti–2wt.% Mo and Ti–18wt.% Mo alloys were prepared from pure components (i.e., 99.98% Ti, 99.99% Nb and 99.97% Mo) by induction melting in an atmosphere of pure argon in the form of 10 mm diameter cylindrical ingots. Then, they were cut by spark erosion into 0.7 mm thin slices. The slices were individually sealed in quartz ampoules with a remaining pressure of 10^−4^ Pa. The quartz ampoules were annealed at 600 and 520 °C for 168 h (for Ti–3wt.% Nb and Ti–20wt.% Nb alloys, respectively) as well as at 1000 °C for 24 h for Ti–2wt.% Mo and Ti–18wt.% Mo alloys. These temperatures of annealing belong to the (α)- and (α + β)-phase field for the Ti–3wt.% Nb and Ti–20wt.% Nb alloys, respectively, as well as to the (β)-phase field for the Ti–2wt.% Mo and Ti–18wt.% Mo alloys (Figure 1). After annealing, the samples were water quenched (together with the ampoules). The chemical compositions of the obtained alloys are presented in a Table 1. The chemical composition was measured by an energy-dispersive X-ray (EDS) spectrometer in scanning electron microscopy. Each value in Table 1 is an average of three measurements.

The annealed samples were HPT-treated at a pressure of 7 GPa, with five anvil rotations at a speed of 1 rpm. The Bridgman anvil-type custom-built computer-controlled device was used produced by W. Klement GmbH, Lang, Austria. The crystal structure and phase composition of the examined alloys were studied with high-energy X-ray diffraction measurements using the beamline P07B (87.1 keV, λ = 0.0142342 nm) at DESY in Germany, Hamburg. Diffraction patterns for the phase analysis were registered in the so-called continuous mode using the area Mar345 Image Plate detector. The experimental setup for phase analyses included rotation by 180 degrees about the ω-axis to avoid the effect of crystallographic texture [33,34]. Such a procedure significantly improves grain statistics by providing quasi-powder diffraction. This, in turn, enables much more accurate results in terms of volume fraction calculation. Secondly, the obtained 2D data were converted by the Fit2D software and presented in a graph of relative intensity versus the 2Theta angle. Rietveld refinement and volume fraction calculations were performed using HighScore Plus software. All microstructural studies on the deformed samples were carried out at a distance of half the radius of the HPT disks. Prior inspection of the initial material was performed using the scanning electron microscope (SEM) FEI E-SEM XL30 (manufactured by FEI, Hillsborough, OR, USA). It was equipped with an EDAX Genesis energy-dispersive X-ray (EDS) spectrometer (FEI, Hillsborough, OR, USA). In order to obtain the composition contrast between different phases in the sample, the backscattered electron signal (BSE mode) was chosen for the SEM images. For study of the fracture surface fractography, the secondary electron signal (SE mode) was used. More demanding examinations of the microstructure were carried out by means of a transmission electron microscopy (TEM) with a TECNAI G2 FEG super TWIN (200 kV) instrument (manufactured by FEI, Hillsborough, OR, USA) equipped with the EDS spectrometer produced by EDAX (AMETEK, Inc., Berwyn, PA, USA). For the production of TEM thin foils, the twin-jet polishing was performed using a D2 electrolyte in a Struers machine (Cleveland, OH, USA). For the analysis of spot diffraction, TIA software for the Tecnai microscope was applied. Additionally, the identification of constituent phases was performed with CARINEV3 software. Decoding of diffraction rings is carried out by the calculation of lattice spacing using ProcessDifraction V_8.7.1 Q software [35]. The study of the thermal stability of the HPT-induced ω-phase was carried out using the in situ XRD measurements at the dedicated Anton Paar HTK 1200 chamber for high-temperature measurements using the Panalytical Empyrean X-ray diffractometer (manufactured by the Malvern Panalytical, Malvern WR14 1XZ, UK) with CuKα radiation and an Al_2_O_3_ sample holder for bulk samples. The chamber was evacuated and, subsequently, flushed and filled with high-purity 6 N argon. Samples were then heated at a heating rate of 5 °C/min up to 940 °C. In the temperature range of 40–940 °C, the diffraction patterns were collected with a temperature step of 20 °C. The diffraction angle 2*θ* interval was chosen between 30 and 80°. The patterns were acquired with an angular step of 0.033°. The acquisition time for each pattern was approximately 25 min. The acquisition each pattern was preceded by temperature stabilization over 10 min. During the in situ experiment on the deformed Ti–18wt.% Mo alloy, doubts arose regarding the interpretation of the phases observed upon heating. To resolve these doubts, an additional TEM observation of the microstructure of the deformed state after heating at 620 °C in an in situ XRD chamber was performed. Since the sizes of the sample in the in situ experiment were small (1 × 1 × 10 mm) for the foil preparation by the conventional twin-jet polishing method, the thin foil was prepared by the focused ion beam (FIB) technique with an FIB Quanta 3D using TECNAI FEG microscopy (30 kV) (FEI, Hillsborough, OR, USA).

## 3. Results and Discussion

**Figure 2:**
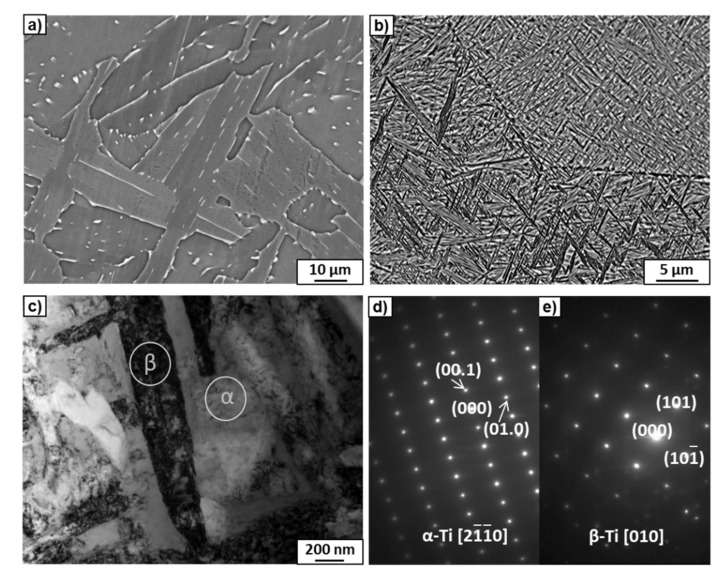
SEM micrographs of the Ti–3wt.% Nb (**a**) and Ti–20wt.% Nb (**b**) alloys annealed at 600 and 520 °C, respectively. TEM bright-field image of Ti–20wt.% Nb (**c**) and the SAED patterns (**d**,**e**) acquired from the areas marked by white circles at the places where α- and β-phases existed, respectively.

**Figure 3:**
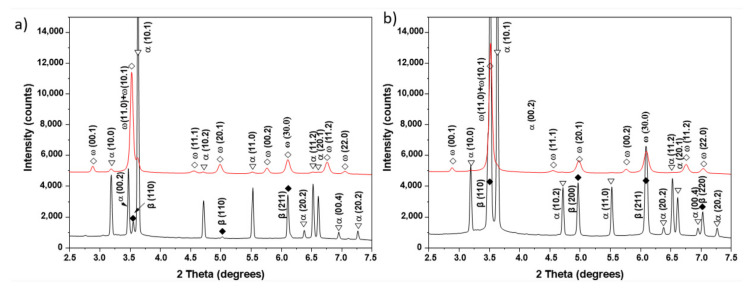
Synchrotron XRD curves of the Ti–3wt.% Nb (**a**) and Ti–20wt.% Nb (**b**) alloys before (black curves) and after HPT (red curves).

Figure 2a,b show SEM micrographs of the coarse-grained initial microstructures of the Ti–3wt.% Nb and Ti–20wt.% Nb alloys after annealing at 600 and 520 °C, respectively. Figure 2a shows the microstructure within one grain, while Figure 2b shows the boundaries of the triple junction of grains. Observation of the microstructure with an optical microscope showed that the grains were round in shape, and their sizes ranged from 0.5 to 1.5 mm. A small amount of uniformly distributed β-phase precipitates could be observed inside the α-phase grain in the case of the Ti–3wt.% Nb alloy (Figure 2a). Since the β-phase grains were enriched in niobium, they had a bright contrast in comparison to the dark α-matrix in the micrographs taken in the BSE mode. In the case of the second alloy, a huge amount of thin (up to 500 nm thick) lamellar precipitates of the β-phase was observed inside the grains of the α-phase (Figure 2b). TEM studies confirmed the presence of the α- and β-phases in the alloys. Examples TEM images of Ti–20wt.% Nb alloy are presented in Figure 2c–e. Selected area electron diffraction (SAED) patterns (Figure 2d,e) were obtained from two different phases marked with white circles in the bright-field image (Figure 2c). The SAED in Figure 2e corresponds to the dark elongated grain of the β-phase, while the SAED in Figure 2e to the α-phase matrix. The EDS microchemical analysis showed enrichment of the α-phase into niobium up to 3wt.% in the case of the Ti–3wt.% Nb alloy and up to 10wt.% in the case of the Ti–20wt.% Nb alloy. According to synchrotron X-ray diffraction analysis (Figure 3), which is described below, the amount of the β-phase was approximately 10% and 40% in the Ti–3wt.% Nb and Ti–20wt.% Nb alloys, respectively (Table 2).

Figure 3 shows synchrotron X-ray diffraction patterns of the examined alloys before and after SPD. Peaks of the α- and β-phases were observed in the initial state of both alloys, wherein only three small peaks of the β-phase were observed in the Ti–3wt.% Nb alloy, and four peaks of this phase appeared in the Ti–20wt.% Nb alloy. After the HPT process, the peaks of the α- and β-phases broadened, which indicates a strong grain refinement of the microstructure and internal stresses in the crystal lattices from the high density of the crystal defects induced by HPT. Peaks of the ω-phase were also observed after HPT, some of which overlapped with the β-phase peaks, but there were some ω-phase peaks (e.g., (00.1), (11.1), (00.2) and (11.2)) that did not overlap with other peaks in the initial state. Since the peaks of the α-phase were significantly reduced in intensity, and some of them disappeared completely after HPT, it can be assumed that the α-phase partially transformed into the ω-phase. Since all peaks of the β-phase observed in the initial state overlapped with the ω-phase peaks after SPD, it is difficult to draw an unambiguous conclusion about the complete β→ω phase transformation.

**Figure 4:**
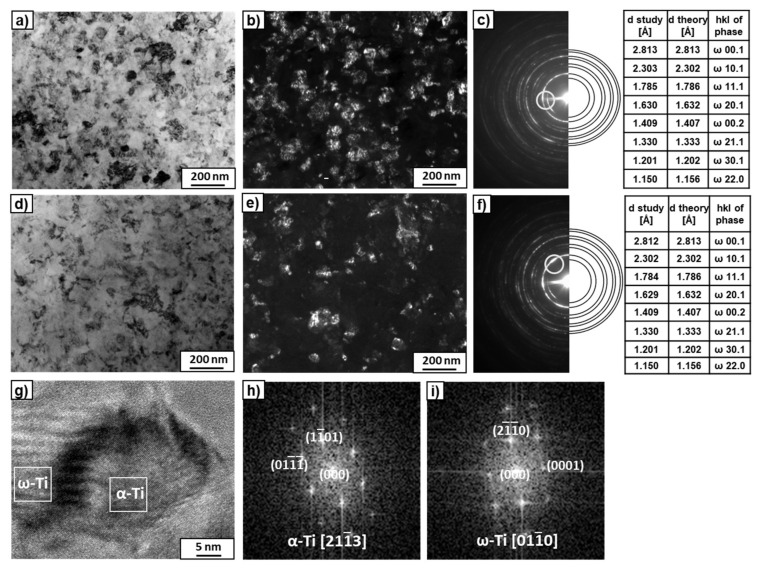
TEM micrographs of the Ti–3wt.% Nb (**a**–**c**) and Ti–20wt.% Nb (**d**–**f**) alloys after the HPT process. Bright-field (**a**,**d**) and dark-field (**b**,**e**) images and the SAED patterns (**c**,**f**) with solutions in the tables. The rings in the SAED patterns indicate the position of the objective aperture for obtaining the dark-field images. A high-resolution TEM image (**g**) of the microstructure of the HPT-deformed Ti–20wt.% Nb alloy with fast Fourier transforms (FFTs) obtained from the marked squares for the α-phase (**h**) and ω-phase (**i**).

TEM studies of the microstructures of the alloys after HPT (Figure 4) showed strong grain refinement up to 50–100 nm. The bright-field (BF) images did not reveal any noticeable differences between the microstructure of the Ti–3wt.% Nb (Figure 4a) and Ti–20wt.% Nb alloys (Figure 4d). On the other hand, the dark-field (DF) image of the Ti–3wt.% Nb alloy (Figure 4b) showed more grains in comparison to the Ti–20wt.% Nb alloy (Figure 4e). This is likely attributed to the fact that for the Ti–3wt.% Nb, the obtained result came from reflexes belonging to two rings of the SAED pattern in the objective aperture (Figure 4c), while for the case of the Ti–20wt.% Nb alloy, reflexes from only one ring were studied (Figure 4f). It is interesting that all rings of the SAED patterns of the deformed alloys fit well with the ω-phase (Figure 4c,f). However, there were some single reflexes (dots) between the recognized rings. Due to the low number of these reflections, the program for determining ring diffraction patterns could not recognize them. It is possible that these single reflections could belong to the α- or β-phases. However, the high-resolution transmission electron microscopy (HRTEM) studies of the thin foils showed the nanograins only in the α- and ω-phases (Figure 4g–i). The HRTEM observations allowed for observation of the separate grains. The columns of atoms were visible when the grain was properly oriented in relation to the electron beam. Figure 4g shows two groups of grains. The measured distances, d_hkl_, and the angles between them in the fast Fourier transforms of the selected grains showed that they belonged to the α- and ω-phases. This implies that the α-phase was present in this sample. A detailed description of the morphology of the α- and ω-phase grains of both examined alloys after HPT can be found in previous works [35,36]. Therefore, it can be concluded that during HPT, the α-phase partially transforms into the ω-phase. Concerning the β-phase, it was found that even a 0.1 plunger rotation during HPT deformation of the Ti–4wt.% Fe alloy in the β-state led to the formation of 90% of the ω-phase [20]. Since the β-phase grains were not detected by the TEM studies of the Ti–3wt.% Nb and Ti–20wt.% Nb alloys, it was concluded that the β-phase completely transformed into the ω-phase during the HPT treatment. Based on synchrotron X-ray diffraction analysis (Figure 3), the volume fraction of the ω-phase was calculated, which amounted to approximately 80% and 86% for the Ti–3wt.% Nb and Ti–20wt.% Nb alloys, respectively (Table 2).

Since the entire β-phase was transformed into the ω-phase, it was easy to calculate (based on the total amount of the ω-phase) the amount of the α-phase that was transformed into the ω-phase. It turned out that this amount was 70% for the Ti–3wt.% Nb alloy and only 46% for the Ti–20wt.% Nb alloy (Table 2). It can be assumed that the more niobium in the initial α-phase (as is observed in the Ti–20wt.% Nb alloy), the more difficult the α→ω phase transformation. This can be explained by an unfavorable change in the lattice parameters, namely, a high content of the alloying element significantly reduces the lattice parameters of the α-phase [35], which makes it difficult for the α→ω phase transformation along certain crystallographic planes during shear deformation induced by HPT. Similar results were also obtained in the Ti–4wt.% Co alloy subjected to HPT under the same conditions [29]. HPT of the Ti–4wt.% Co alloy resulted in a strong grain refinement of the microstructure and a partial α→ω phase transformation. It was found that HPT-induced α→ω phase transformation depended on the cobalt content in the initial α-phase and the morphology of the microstructure. The lower cobalt content and smaller grain size of the α-phase led to a higher amount of ω-phase induced by HPT [29].

**Figure 5:**
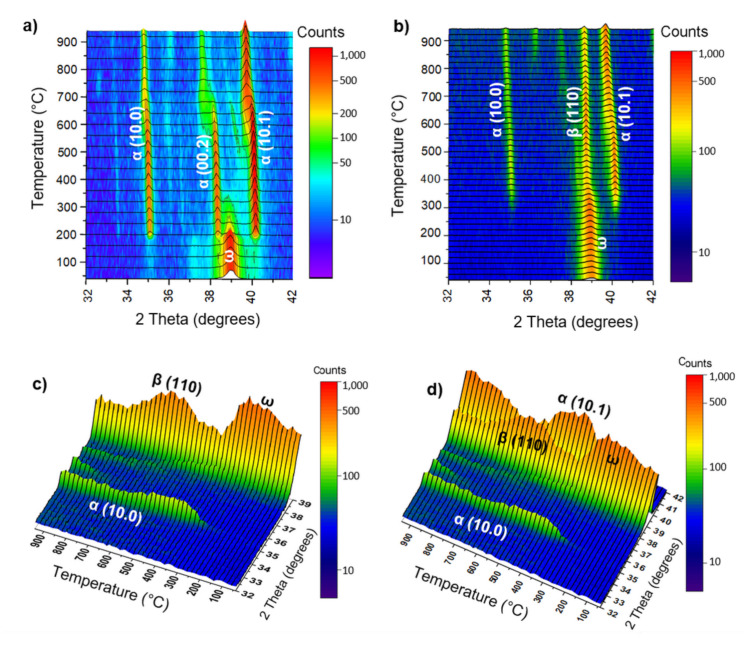
The XRD in situ heating map of the HPT-deformed Ti–3wt.% Nb (**a**) and Ti–20wt.% Nb (**b**–**d**) alloys. The ω-phase is presented by a (11.0 + 10.1) doublet of peaks at the angle of 2θ 39.09°. Lateral projections (**c**,**d**) of the map shown in (**b**) in the 32–39° (**c**) and 32–42° (**d**) range of 2θ angles. Figure 5a is reprinted with permission from Reference [35].

The thermal stability of the metastable ω-phase was studied by in situ high-temperature XRD measurements. The in situ XRD maps of the examined alloys after HPT are presented in Figure 5. As can be seen, only the (11.0) + (10.1) doublet of ω-phase peaks can be distinguished in the 32–42° range of 2θ angles on the maps of both alloys before their heating. However, the (10.0) and (10.1) peaks of the α-phase are clearly visible in this range of 2θ angles on the standard XRD curves in Figure 3. The absence of the α-phase peaks in the 32–42° range of 2θ angles in the in situ XRD map is related to the different durations of the XRD measurements. For the in situ method, there is not enough time to register the many X-ray counts compared to the standard method.

Heating of the Ti–3wt.% Nb alloy above 200 °C resulted in a significant decrease in the intensity of the ω-phase (11.0 + 10.1) doublet of peaks and, simultaneously, in the appearance of well-defined α-phase peaks (10.0), (00.2) and (10.1). This means that the decomposition of the ω-phase into the α-phase ended at a temperature of 270 °C. In the case of the Ti–20wt.% Nb alloy (Figure 5b–d), the decomposition of the ω-phase began at 280 °C and ended at 370 °C, which is clearly seen on the lateral projection of the XRD in situ map (Figure 5c). A distinctive feature of ω-phase decomposition in this alloy is the appearance of the α- and β-phases. The intensity of the β-phase peak (110) first increases during heating to 630 °C and then decreases, while the intensity of this peak does not exceed the intensity of the α-phase peak (10.1) (Figure 5d). It should be noted that when both alloys were heated above 600–650 °C, the α-phase peaks shifted slightly to the left side (towards the lower diffraction angles), which is associated with an increase in the lattice parameters of the α-phase. Since the ω-phase is enriched in niobium as an alloying element (by analogy with the ω-phase enriched in iron in Ti–Fe alloys [17,30]), it was assumed that the α-phase arising after the decomposition of the ω-phase was also enriched in niobium. Therefore, a slight shift in the α-phase peaks towards lower diffraction angles at heating to the highest temperatures can be associated with the appearance of the new α-phase with lower niobium content as well as the increase in the lattice parameters due to the thermal expansion.

Comparing the decomposition temperatures of the ω-phase of both examined alloys, it can be seen that the thermal stability of the ω-phase in the Ti–20wt.% Nb alloy was slightly higher (Table 2). Perhaps this was due to the higher content of the alloying component in the ω-phase of this alloy. Since the ω-phase is formed in a diffusionless way, this means that its chemical composition will coincide with the chemical composition of the phase from which it formed. In the Ti–20wt.% Nb alloy, the amount of the β-phase converted to the ω-phase was higher. In addition, the β- and α-phases in this alloy were more enriched in niobium than these phases in the Ti–3wt.% Nb alloy (Table 2). For example, the β-phase in the Ti–20wt.% Nb alloy contained 50wt.% Nb, but in the Ti–3wt.% Nb alloy only 15wt.% Nb. This means that the ω-phase in the Ti–20wt.% Nb alloy was more enriched in niobium.

**Figure 6:**
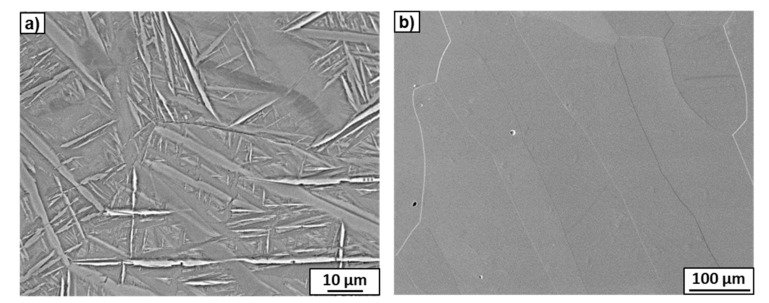
SEM micrographs of the Ti–2wt.% Mo (**a**) and Ti–18wt.% Mo (**b**) alloys after annealing at 1000 °C.

**Figure 7:**
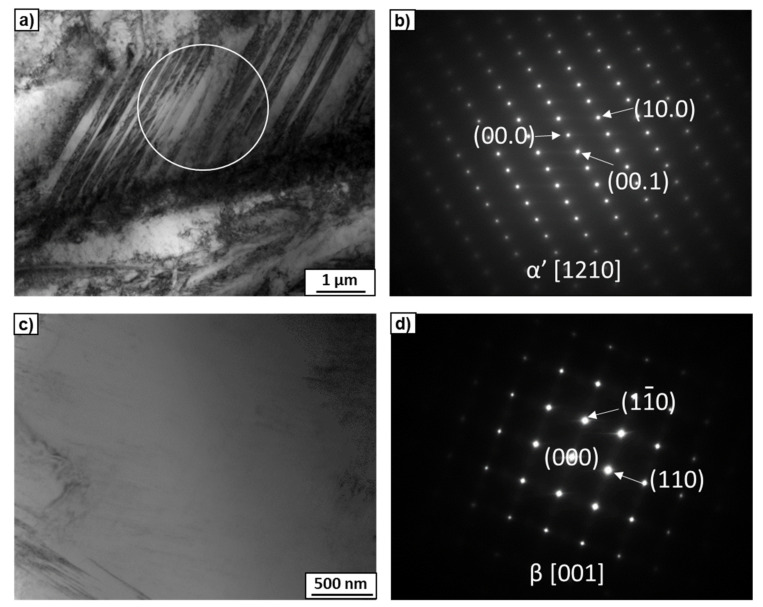
TEM micrographs of the Ti–2wt.% Mo (**a**,**b**) and Ti–18wt.% Mo (**c**,**d**) alloys after annealing at 1000 °C. Bright-field images (**a**,**c**) with the SAED patterns (**b**,**d**) indicate α′- and β-phases. The white circle marked in (**a**) shows the area from which the SAED pattern was obtained.

The microstructures of the Ti–2wt.% Mo and Ti–18wt.% Mo alloys after annealing at 1000 °C are shown in Figure 6. According to the equilibrium diagram, both alloys must contain a β-solid solution. However, the microstructure of the Ti–2wt.% Mo alloy showed the thin plates inside large grains (Figure 6a). The TEM study of the thin foil showed that the morphology of the plates was close to martensite, which was confirmed by the SAED patterns (Figure 7a,b). It is possible that the cooling rate of the sample after annealing was not high enough to maintain the β-phase, which decomposed to α′-martensite with hexagonal close-packed (hcp) unit cells. The microstructure of the Ti–18wt.% Mo alloy consisted only of large elongated grains of the β-phase (Figure 6b and Figure 7c,d). In spite of the same cooling rate, α′-martensite was not present, since according to the Ti-Mo system, it can only appear at an Mo content of up to 2–3 at.% [14].

**Figure 8:**
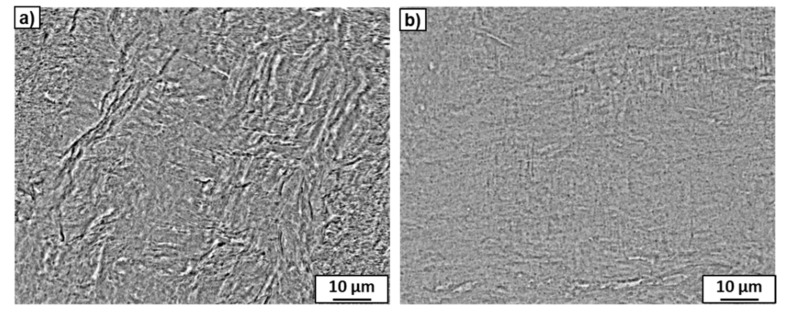
SEM micrographs of the Ti–2wt.% Mo (**a**) and Ti–18wt.% Mo (**b**) alloys after HPT.

**Figure 9:**
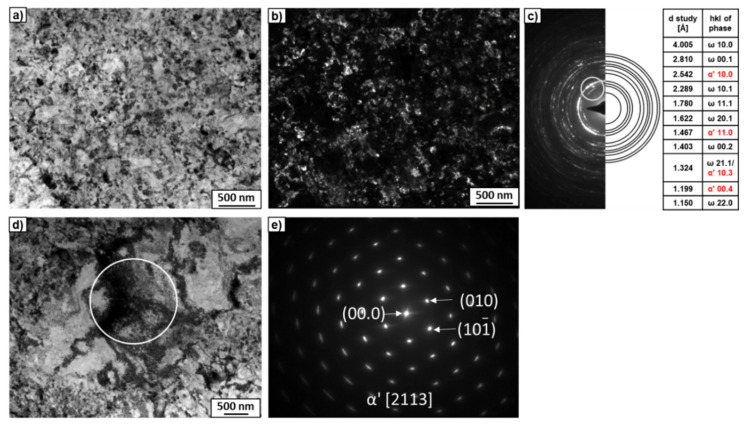
TEM micrographs of the Ti–2wt.% Mo after HPT. Bright-field images (**a**,**d**) and a dark-field image (**b**) with the SAED patterns (**c**,**e**). The white circle marked in (**d**) shows the area from which the SAED pattern (**e**) was obtained. The ring in the SAED pattern indicates the position of the objective aperture for obtaining the dark-field image.

The SEM micrographs of both alloys after HPT are shown in Figure 8. It can be seen that the microstructure of the Ti–2wt.% Mo alloy was less homogeneous compared to the Ti–18wt.% Mo alloy: martensite plates are visible within the surrounding matrix (Figure 8a). The detailed studies using TEM showed a strong refinement of the microstructure containing a mixture of α′- and ω-phase nanograins (Figure 9e) and a single grain of the α′-martensite with a high dislocation density. It is possible that some plates of the α′-martensite in the initial state were unfavorably oriented for the activation of the slip systems responsible for the phase transformation, and the increased dislocation density in these plates after HPT was insufficient to initiate the α′→ω phase transformation.

**Figure 10:**
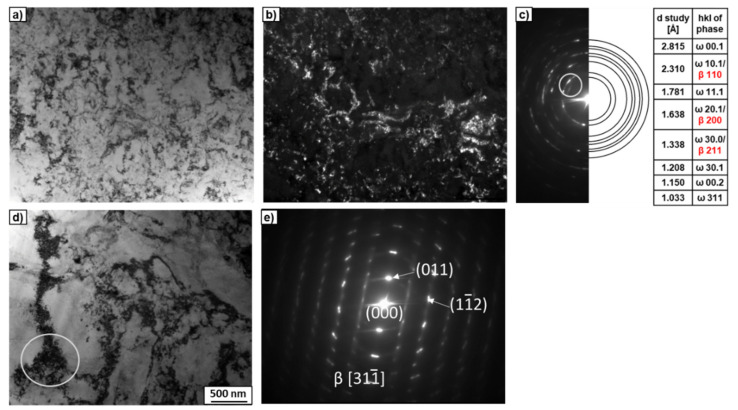
TEM micrographs of the Ti–18wt.% Mo after HPT. Bright-field image (**a**) and dark-field image (**b**) with the SAED pattern (**c**). The ring in the SAED pattern (**c**) indicates the position of the objective aperture for obtaining the dark-field image. The white circle marked in (**d**) shows the area from which the SAED pattern (**e**) was obtained.

The microstructure of the Ti–18wt.% Mo alloy after HPT was not homogeneous, with the presence of fine-grained (Figure 10a–c) and coarse-grained (Figure 10d,e) regions. However, even fine-grained areas seemed to be coarser compared to the Ti–2wt.% Mo alloy. This was evidenced by a smaller number of SAED reflexes (Figure 10c), taken from the region of the same size as in the case of the Ti–2wt.% Mo alloy. Most of the SAED reflexes in Figure 10c belong to the ω-phase, but there were also a few reflexes that coincided with the β- and ω-phases simultaneously. In the SAED pattern taken from the coarser-grained area in Figure 10d, an overlap of at least two crystal lattices can be seen, one of which fits well with the β-phase (Figure 10e).

**Figure 11:**
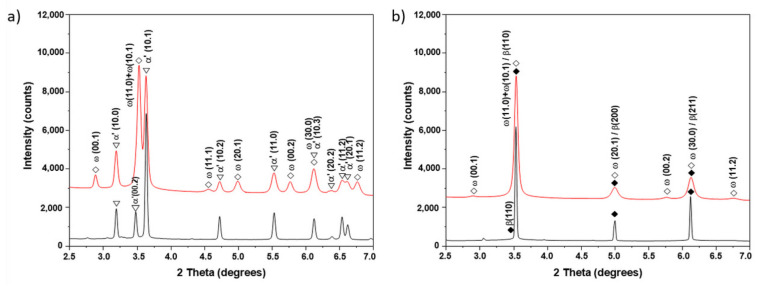
Synchrotron XRD curves of the Ti–2wt.% Mo (**a**) and Ti–18wt.% Mo (**b**) alloys before (black curves) and after HPT (red curves).

Synchrotron XRD analysis (Figure 11) confirmed that the initial states of the Ti–2wt.% Mo and Ti–18wt.% Mo alloys were α′-martensite and β-solid solution, respectively. After deformation, a significant broadening of all peaks and the appearance of ω-phase peaks are observed. The presence of the high-pressure ω-phase in both alloys indicates the partial α′→ω and β→ω phase transformations occurred. The amount of ω-phase reached about 65.6% in the Ti–2wt.% Mo, and only 47.6% in the Ti–18wt.% Mo alloys.

When analyzing the phase composition of the deformed Ti–18wt.% Mo alloy, the following question may arise: Why did the β-phase, which has a large dimensional and structural correspondence with the ω-phase in this alloy, not transform completely into the ω-phase (as in the case of the Ti–20wt.% Nb alloy)? It can be assumed that this was due to the morphology of the initial microstructures of both alloys. The Ti–20wt.% Nb is a two-phase alloy and contains small plates of α- and β-phases in the initial state, while the Ti–20wt.% Nb alloy is a coarse-grained single-β-phase solid solution. It is known that, for a phase transformation, the original phase must overcome a certain energy barrier associated with the accumulation of stresses in the microstructure [25]. It is possible that a two-phase microstructure (or a single-phase microstructure with a fine grain size) piles up deformation more easily compared to a single-phase coarse-grained structure due to the presence of a large fraction of interphase boundaries (grain boundaries), which are obstacles for the movement of dislocations. Therefore, it is possible that the small α- or β-plates overcome the critical shear stresses necessary for the phase transformations and transform into the ω-phase faster in comparison to the single-phase coarse-grained structures. Similarly, the presence of a fine lamellar martensitic structure in the initial state of the Ti–2wt.% Mo alloy explains the formation of a greater amount of the ω-phase compared to the coarse-grained single-β-phase of the Ti–18wt.% Mo alloy.

**Figure 12:**
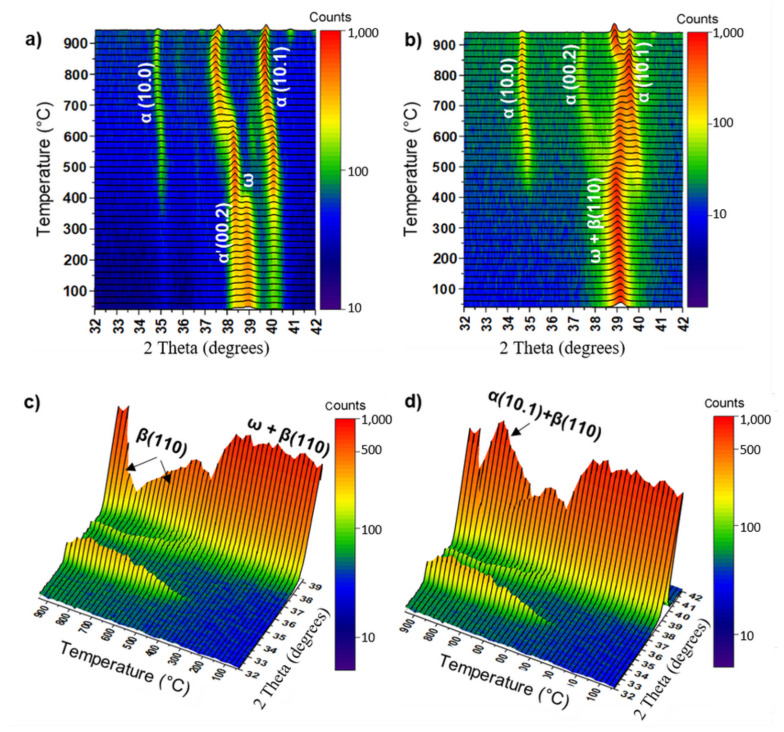
The XRD in situ heating map of the HPT-deformed Ti–2wt.% Mo (**a**) and Ti–18wt.% Mo (**b**–**d**) alloys. The ω-phase is presented by a (11.0 + 10.1) doublet of peaks at the angle of 2θ 39.09°. Lateral projections (**c**,**d**) of the map are shown in (**b**) in the 32–39° (**c**) and 32–42° (**d**) range of 2θ angles.

The results of the studies of the ω-phase thermal stability in the deformed Ti–2wt.% Mo and Ti–18wt.% Mo alloys are shown in Figure 12. Three peaks belonging to α′(00.2), ω(10.1)/(11.0) and α′(10.1) can be seen on the XRD in situ heating map of the deformed Ti–2wt.% Mo alloy in the 2θ angle range of 32–42° before its heating (Figure 12a). Heating of the alloy above 300 °C leads to a gradual decrease in the intensity of the ω-phase peak. The ω-phase peak disappeared at a temperature of approximately 400 °C. At the same time, the intensity of the α′(00.2) and α′(10.1) peaks increased, and a new α′(10.0) peak appeared. All this testifies to the decomposition of the ω-phase into the α′- or α-phase. When heated above 600 °C, a shift in the α′-phase peaks towards lower angles was observed. This shift indicates an increase in the lattice parameters. This increase can be a result of lattice thermal expansion from the heating. It should be noted that, theoretically, the α′-martensite should be more enriched in Mo than the α-phase, since α′-martensite comes from the β-phase enriched in Mo due to the diffusionless transformation. Therefore, the lattice parameters of α′- martensite should be slightly smaller than that of the α-phase, and their peaks should be somewhat shifted towards higher angles of 2θ. But, in the case of the Ti–2wt.% Mo alloy, the low content of Mo means the lattice parameters of the α′-martensite and the α-phase are almost the same (for α′-phase *a* = 2.949 ± 0.006 nm, *c* = 4.681 ± 0.006 nm; for α-phase: *a* = 2.954 ± 0.006 nm, *c* = 4.690 ± 0.006 nm), so there is no visible difference in peak positions between these two phases. It should be noted that, in the case of the Ti–1wt.%Fe alloy in the initial state of α′-martensite and a small amount of β-phase, an assumption was made about the probable α′→α→ω sequence of transformations during HPT deformation [30]. The authors of this work noticed a significant increase in the lattice parameters of the α′ after HPT, and their approach to the parameter of pure titanium (α) already after a 0.1 anvil revolution, when the ω-phase had not yet formed. This result led to the assumption that, at the first stage of deformation, the α′-phase transforms into the α-phase which, in turn, transforms into the ω-phase.

One peak at the 2θ angle of 39.1° was observed in the XRD in situ heating map before heating of the deformed Ti–18wt.% Mo alloy (Figure 12b). This peak corresponded to the ω- (11.0 + 10.1) and β- (110) phases. It can be seen that this peak slightly shifted to the right compared to the peak in the Ti–2wt.% Mo alloy. This is explained by a decrease in the lattice parameter of the ω-phase in the Ti–18wt.% Mo alloy due to the higher content of molybdenum (Table 3). With the heating of the Ti–18wt.% Mo alloy, the intensity of this peak decreased in the temperature range of 420–500 °C (Figure 12c), and formation of new peaks from the α-phase were observed at the same time. This indicates the decomposition of the ω-phase. Shifting the peak of the β-phase (110) towards larger angles after the decomposition of the ω-phase is associated with the enrichment of the β-phase with molybdenum. It is possible that molybdenum from the decomposed ω-phase diffused into the β-phase, but when heated above 850 °C, the chemical composition of the β-phase came into an equilibrium state, and the peak of the β-phase shifted again to the left. To check whether the shifted peak of the β-phase after the decomposition of the ω-phase really belonged to the β-phase, the deformed sample was annealed in an in situ XRD chamber at a temperature of 620 °C for 10 min and then a thin foil was made using the FIB method.

**Figure 13:**
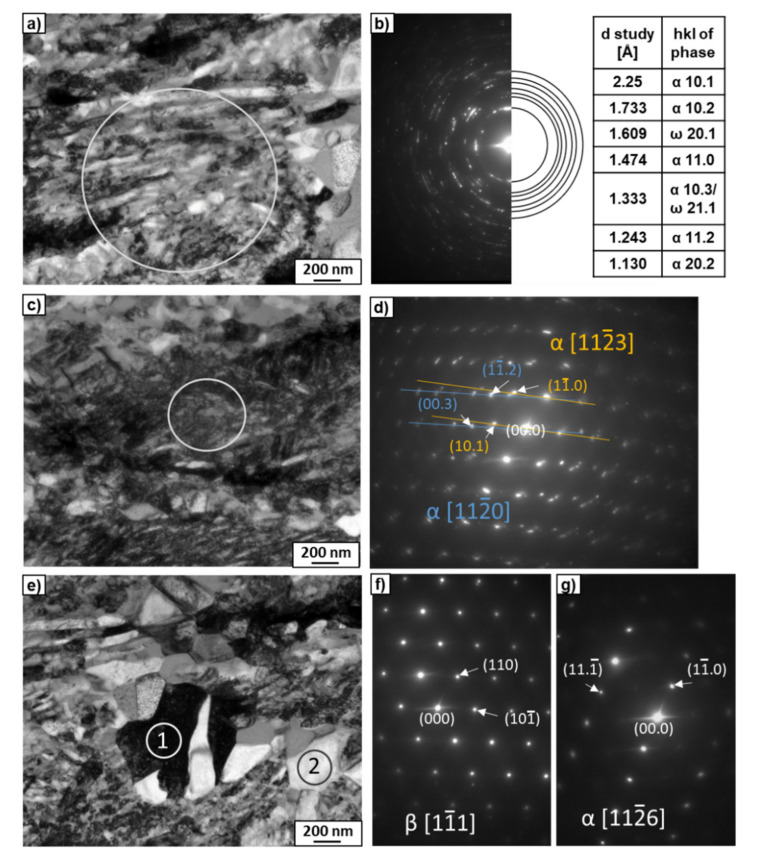
TEM micrographs of the Ti–18wt.% Mo after HPT and heating at 620 °C. Bright-field images (**a**,**c**,**e**) with SAED patterns (**b**,**d**,**f**,**g**). SAED patterns (**b**,**d**) were taken from the areas marked by rings in (**a**,**c**), respectively. The SAED patterns (**f**,**g**) were taken from grains marked as 1 and 2 in (**e**), respectively.

The TEM studies of a thin foil of the Ti–18wt.% Mo alloy after HPT and heating at 620 °C (Figure 13) showed the microstructure including regions with a fine-grained microstructure (Figure 13a,b), coarse-grained regions with an increased density of dislocations (Figure 13c,d) and large grains (up to 500 nm in size) with a regular shape and free of dislocations (Figure 13e–g). Most of the diffraction reflexes in the fine-grained area (Figure 13a) belonged to the α-phase, one belonged to the ω-phase, and the other was common to both phases (Figure 13b). The presence of only one unambiguous reflection of the ω-phase indicates its almost complete decomposition. The coarse-grained regions with an increased density of dislocations corresponded also to the α-phase. Figure 13d shows the SAED pattern, which is an overlay of two lattices of the α-phase. The SAED patterns taken from areas 1 and 2 in Figure 13e showed the presence of the β- (Figure 13f) and α-phases (Figure 13g), respectively. The EDS microchemical analysis showed that the grains of the α-phase were enriched in Mo from 0.1 to 0.9wt./%, while the grains of the β-phase from 28.3 to 33.4wt.% Mo. The high content of Mo in the β-phase observed after the ω-phase decomposition (compared to the initial β-phase with a Mo content of 18 wt.%), explains the shift in the peak of the β-phase (110) towards higher 2θ angles (Figure 12b).

When comparing the Ti–18wt.% Mo alloy with the Ti–20wt.% Nb alloy, the following question arises: Why does the position of the β-phase peak (110) in the alloy with niobium not shift to the right after the decomposition of the ω-phase? The point is that after HPT of the Ti–20wt.% Nb alloy, the β-phase completely transformed into the ω-phase. Under the heating of the deformed Ti–20wt.% Nb alloy, the ω-phase decomposed into the α- and β-phases, and the β-phase immediately obtained the required chemical composition close to equilibrium. In the case of the Ti–18wt.% Mo alloy, the β-phase was already present in the microstructure before the decomposition of the ω-phase, and when the ω-phase decomposed, the molybdenum atoms, as a beta stabilizer, passed to the β-phase and enriched it which, in turn, resulted in a decrease in its lattice parameters and, therefore, to a slight shift in the position of the β-phase peak (110).

It was shown for the alloys belonging to the Ti-Nb and Ti-Mo systems that the formation of a high-pressure ω-phase may depend on the phase composition and grain size of the initial microstructure. In the case of the Ti–Nb alloys, it was found that an increase in the alloying element in the α-phase hinders the process of the α→ω phase transition. In [29], using a Ti–4wt.% Co alloy deformed by the HPT, this phenomenon is explained by a decrease in the lattice parameters of the α-phase, which is unfavorable for its transition to the ω-phase. The ω-phase is more easily formed from the β-phase due to the large dimensional and structural similarities between these phases; therefore, in the studied two-phase (α + β) Ti–Nb alloys, a complete β→ω phase transformation can be seen. However, in the case of the Ti–18wt.% Mo alloy with the initial β-phase coarse-grained microstructure, only 47% of the β-phase underwent phase transformation. In this case, the amount of the obtained ω-phase was less than the amount of the ω-phase formed from the finely dispersed martensitic microstructure of the deformed Ti–2wt.% Mo alloy. It is possible that the size of the grains also influenced the phase transformation, since it is easier for small plates (grains) to gain deformation and overcome the critical shear stresses necessary for α→ω or β→ω transformations.

Comparing the thermal stability of the ω-phase in the studied Ti–Nb and Ti–Mo alloys, it can be seen that an increase in the alloying element in the ω-phase leads to an increase in its thermal stability from 200 to 370 °C for Ti–Nb alloys and from 300 to 500 °C for Ti–Mo alloys. The formation of the high-pressure ω-phase is martensitic (diffusionless), but is the decomposition of the ω-phase also diffusionless? This question is open, because the mechanism of this transformation is still not fully understood and not sufficiently documented in the literature. There are suggestions that the decomposition of the ω-phase may be of a diffusion nature. In [29], the thermal stability of the ω-phase formed from the α-solid solution of HPT-deformed Ti–Co alloys was studied using different scanning calorimetry. It turns out that the temperature interval of the reverse-phase transformation ω→α varied depending on the heating rate: the lower the heating rate, the greater the shift in the temperature interval towards lower temperatures. This dependence indicates the diffusion nature of this transformation or at least the diffusion contribution to the transformation. In addition, it was shown in [37] that the reverse transformation ω→α is prevented by crystal defects in the ω-phase. A significant decrease in the dislocation density in the ω-phase before the initiation of the ω→α transformation was found. Since dislocation annihilation is a thermally activated process, this makes a significant contribution to the observed phase transformation. Thus, if the process of the reverse-phase transformation has a diffusive character, then the decomposition of the ω-phase with a high content of the alloying element may require a larger amount of diffusive mass transfer, which is achieved by increasing the heating temperature of the deformed material. One can assume that a ω-phase with a high content of the alloying element will contain more defects in the crystal structure caused by SPD. The annihilation of these defects with an increase in temperature will promote the decomposition of the ω-phase. It should be noted that alloying titanium with beta-stabilizing elements, such as Nb and Mo as well as Fe [28] and Co [29], leads to a significant increase in the thermal stability of the ω-phase compared to pure titanium supplied by HPT, where the process of decomposition of the ω-phase was observed in a temperature range of 140–180 °C [17]. It should be noted that knowledge on the thermal stability of the ω-phase is necessary to determine the conditions for the thermo-mechanical treatment of Ti alloys in order to modify their mechanical properties. It follows from a review of the literature that the use of short-term, low-temperature annealing before tensile testing [38,39] or tension at elevated temperatures [24,40] can lead to an improvement in the plasticity of HPT-deformed alloys. For example, the short-term annealing of HPT-treated titanium at 200–300 °C increased both the ductility and strength [38,39]. Since the metastable ω-phase in the pure Ti remained in the HPT-deformed samples only up to 180 °C [17], the observed changes in the mechanical properties under annealing could be due to the ω→α back transformation. The important increase in ductility together with a slight strength decrease was also observed in the Zr-2.5wt.% Nb alloy after HPT and low-temperature annealing [41], where these changes in the properties were correlated with a partial reverse ω→α phase transformation. Therefore, the application of the HPT process and additional annealing is very promising for the development of advanced metallic biomaterials with high levels of mechanical properties. Currently, a small number of works are dedicated to the influence of the ω-Ti on the mechanical behavior of the SPD-treated alloys, where generally the hardness and Young’s modulus are usually measured [24,25,36,41,42,43], while there are practically no data on the tensile strength. The next step of our work will be to study the effect of the α→ω and ω→α phase transformations on the mechanical properties (i.e., strength and ductility) of alloys after HPT and after HPT with low-temperature annealing, determined in a tensile experiment.

## 4. Conclusions

The effect of HPT deformation on the microstructure and phase transformations of the Ti–2wt.% Nb, Ti–20wt.% Nb, Ti–2wt.% Mo and Ti–18wt.% Mo alloys with different phase compositions (i.e., (90%α + 10% β), (60%α + 40%β), (100%α′) and (100%β), respectively) was examined. It was shown that the formation of the high-pressure ω-phase depended on the phase composition, chemical composition of the phases and the grain size of the initial microstructure. An increase in the content of the alloying element in the α-phase in two-phase (α + β) Ti–Nb alloys complicates the process of α→ω phase transition due to the distortion of the crystal lattice of the α-phase. At the same time, in these two-phase alloys, complete β→ω phase transformation was observed. In the case of single-phase alloys of the Ti–Mo system, the high-pressure ω-phase was shown to be more difficult to form from the coarse-grained β-phase (Ti–18wt.% Mo alloy) than from fine α′-martensite (Ti–2wt.% Mo alloy). It is possible that it is easier for the small plates to deform and overcome the critical shear stresses necessary for phase transformation.

It was also found that the thermal stability of the ω-phase in the Ti–Nb and Ti–Mo alloys depended on the content of the alloying element in this phase, namely, the higher the content of the alloying element, the higher the thermal stability of the ω-phase. It is possible that this was due to the diffusion nature of the decomposition of the ω-phase. For the decomposition of the ω-phase with a high content of the alloying element, a larger volume of mass transfer due to the diffusion process is required, which is achieved by increasing the heating temperature of the deformed material. It should be noted that alloying titanium with β-stabilizing elements (i.e., Nb, Mo, Fe and Co) leads to a significant increase in the thermal stability of the ω-phase compared to pure titanium.

## Figures and Tables

**Table 1:** The chemical composition of the investigated alloys.

Ti–3wt.% Nb	Ti–20wt.% Nb	Ti–2wt.% Mo	Ti–18wt.% Mo
3.1 ± 0.5wt.% Nb 96.9 ± 1.9wt.% Ti	19.7 ± 0.8wt.% Nb 80.3 ± 1.6wt.% Ti	1.9 ± 0.9wt.% Mo 89.1 ± 1.8wt.% Ti	17.8 ± 0.7wt.% Mo 82.2 ± 1.6wt.% Ti

**Table 2:** The volume fraction of some phases before and after HPT (measured by synchrotron analysis), the Nb content in the α- and β-phases in the initial state and the thermal stability of the ω-phase (measured by the XRD in situ method). The chemical compositions of the α- and β-phases were measured by TEM studies.

Alloy	Before HPT			After HPT		
	Phase Composition of the Initial State, %	Nb Content in the α-Phase, wt.%	Nb Content in the β-Phase, wt.%	Volume Fraction of the ω-Phase after HPT, %	Volume Fraction of the α-Phase Transformed to the ω-Phase, %	Thermal Stability of the ω-Phase, °C
Ti–3wt.% Nb	90α + 10β	3.0 ± 0.4	15.0 ± 2.2	80 ± 5	70 ± 5	200–270
Ti–20wt.% Nb	60α + 40β	10.0 ± 1.5	50.0 ± 7.5	86 ± 5	46 ± 5	280–370

**Table 3:** The volume fraction and lattice parameters of some phases before and after HPT as the thermal stability of the ω-phase measured by the XRD in situ method.

Alloy	Before HPT		After HPT			
	Phase Composition, %	Lattice Parameters, nm	Phase Composition, %	Lattice Parameters of α′/α- or β-Phases, nm	Lattice Parameters of ω-Phase, nm	Thermal Stability of ω-Phase, °C
Ti–2wt.% Mo	100% α′	*a* = 0.2949 *c* = 0.4681	35 ± 5% α′ + 65 ± 5% ω	*a* = 0.2949 *a* = 0.4686	*a* = 0.4626 *a* = 0.2827	300–400
Ti–18wt.% Mo	100% β	*a* = 0.3250	53 ± 5% β + 47 ± 5% ω	*a* = 0.3256	*a* = 0.4620 *a* = 0.2821	420–500
Pure Ti [20]	100% α	*a* = 0.2954 *c* = 0.4690	60 ± 5% α + 40 ± 5% ω	*a* = 0.2959 *a* = 0.4690	*a* = 0.4627 *a* = 0.2830	140–180 [17]

## Data Availability

The data presented in this study are available on request from the corresponding author.
